# Impact of intergenerational support and medical expenditures on depression: Evidence from rural older adults in China

**DOI:** 10.3389/fpubh.2022.840864

**Published:** 2022-07-22

**Authors:** Congrong Li, Qing Han, Jinrong Hu, Zeyu Han, Hongjuan Yang

**Affiliations:** ^1^School of Public Administration, Xi'an University of Architecture and Technology, Xi'an, China; ^2^Institute of Sociology, Shaanxi Academy of Social Sciences, Xi'an, China

**Keywords:** older adults, depression, intergenerational support, medical expenditures, rural

## Abstract

**Objective:**

Globally, depression has become a major health issue among older adults, who experience poor physical health and high medical expenditures. In Asian countries, older adults are greatly dependent on their children. This study assessed the impact of different types of intergenerational support and medical expenditures on depression among older adults in rural China.

**Method:**

A three-phase balanced panel was constructed based on data from 1,838 rural older adults with comparable scores on the Center for Epidemiologic Studies Depression Scale (CES-D) from the China Family Panel Studies in 2012, 2016, and 2018. A fixed-effects model was used to analyze the impact of intergenerational support and medical expenditures on CES-D score and of intergenerational support on medical expenditures. The propensity score-matching model was used to test the regression results' robustness.

**Results:**

The findings were as follows. First, different types of intergenerational support had a heterogeneous impact on depression among rural older adults. Emotional support had a significantly negative impact on CES-D score, although too much care-based support had a positive impact on CES-D score. Low-level economic support had no significant effect on CES-D score. Second, medical expenditures impacted depression; among these, non-inpatient medical expenditure had a significant and positive impact on CES-D score. Third, CES-D scores among rural older adults were associated with chronic diseases and per capita family income. Fourth, care-based support was associated with reduced non-inpatient medical expenditures, and the sub-sample regression results indicated that the impact was significant for older adults with no chronic diseases and those younger than 75 years.

**Conclusion:**

Intergenerational emotional support and non-inpatient medical expenditures directly affected rural older adults' CES-D scores. The mediating role of medical expenditures between intergenerational support and CES-D score was not significant. Measures should be taken to encourage intergenerational emotional support and reduce the pressure on children's economic and care-based support. Further, the medical insurance reimbursement policy, as formal support, should be improved to alleviate depression among rural older adults when children's support is limited.

## Introduction

Based on data from China's seventh census, approximately 23.81% of rural residents are older adults, which is 7.99% higher than for residents of cities and towns. As aging is a constant process, the prevalence of psychological diseases such as dementia, depression, and anxiety among older adults is also rising (1). According to the World Health Organization's statistics, 15% of older adults have a mental disorder, the most common of which are depression and anxiety (2). Of the total suicides among Chinese older adults, 36% of cases involve psychological issues. Geriatric depression has also emerged as a serious health problem, particularly in low- and-middle-income countries and regions (3). For instance, in some rural areas of India, the prevalence of depression among older adults is over 60% (4). In China, almost half of rural older adults have depressive symptoms (5), and the suicide rate among older adults in rural areas owing to psychological problems is approximately 1.83 times higher than that in urban areas (6). In this regard, psychological health issues among rural older adults require urgent social attention.

Social support exerts a significant influence on older parents' psychological well-being, particularly in East Asian societies that are under the influence of traditional norms such as filial piety; these include China (7), South Korea (8), and Thailand (9). Children are the core actors in family support, especially in rural areas, where social services for older adults are still in the nascent stage. Older adults are generally unlikely to accept institutional care (10); thus, intergenerational support has a great impact on older adults in rural areas. The need for medical services for older adults also increases as physical health worsens with age (11); catastrophic health payments are common in low-income regions (12), increasing the financial and psychological burden. Owing to a loss of labor ability and lack of income sources, older adults are more likely to drop out of treatment (13). Additionally, while China's New Rural Cooperative Medical System (NRCMS) has reduced immediate outpatient expenses, they remain expensive (14). Thus, older adults still rely on their children for care, company, and financial support.

Existing studies have indicated that a deteriorating physical condition aggravates depression in older adults (15). This raises an interesting question: would an increase in medical service expenditures, which closely correspond to physical health, also aggravate depression in older adults? As adult children are at the core of family support, would different types of intergenerational support provided by children help improve mental health among rural older adults? Additionally, would intergenerational support impact older adults' mental health by eliminating concerns regarding medical expenditures? To answer these questions, the current study proposes that three factors—intergenerational support, medical expenditures, and depression among older adults—be combined into one research framework. Specifically, in this study of rural older adults, we analyze the impact of intergenerational support and medical expenditures on depression among rural older adults and the impact of intergenerational support on medical expenditures. We further examine sample heterogeneity through a sub-sample regression based on different health conditions and age groups among older adults. Overall, this study explores the role of intergenerational support while actively addressing aging.

## Literature review and hypotheses

### Intergenerational support and mental health among older adults

With age, physical health problems and social isolation become increasingly prominent, and mental health is affected by intergenerational and social support, among other factors (16). In the context of China, as adult children and spouses are the primary caregivers (17), the relationship between depression and family support becomes particularly significant for older adults (18). Intergenerational support manifests as economic, care-based, and emotional support from children (19, 20). Economic support refers to financial or material support, care-based support refers to the support provided in housework and daily life, and emotional support refers to the intimate relationships generated through communication. Children bear the main responsibility of support, and intergenerational support inevitably impacts health among older adults. However, no consensus exists as to whether intergenerational support can promote mental health in older adults.

The literature posits that intergenerational support helps promote older adults' mental health (21). Some scholars have proven that children's economic support can alleviate the pressure on older adults, provide a sense of security, and promote their mental health (22). Life care helps enhance older adults' life satisfaction and mental health (23) and reduces the incidence of depression by increasing opportunities to participate in social activities and exercise (24). Emotional interaction can reduce loneliness among older adults and improve their life satisfaction and mental health (25, 26). In this regard, the emotional comfort that children offer older adults within a household is irreplaceable.

Other studies have reported contradictory findings, such as that while economic support is related to life satisfaction, it does not have a significant impact on depression (27). Certain scholars have posited that intergenerational support improves older adults' physical and mental health. Children's economic and care-related support has been reported to lead to a decline in older adults' self-care ability (28), in that daily care becomes a burden for older adults and reduces their self-efficacy, thus further aggravating depression (29). Continuous care can create discontent that leads to intergenerational conflicts, reducing children's willingness to provide care and diminishing the effects of that care, which is not conducive to improving mental health among older adults (30).

Based on the above discussion, whether intergenerational support promotes or inhibits depression among older adults largely depends on what older adults actually need. Rural socialized services for older adults and medical resources are still developing (31), and rural older adults are generally weak in terms of social security and pensions. Additionally, owing to the stigma associated with living in care homes in most Asian countries (32), nearly all (93%) older parents expect their children to take care of them (33), and children's support has a great impact on older adults' health. Therefore, the current study proposes the following hypotheses:

H1: Intergenerational support has a negative impact on depression among rural older adults.

As intergenerational support is divided into three types, it is proposed that:

H1a: Economic support has a negative impact on depression among rural older adults.

H1b: Care-based support has a negative impact on depression among rural older adults.

H1c: Emotional support has a negative impact on depression among rural older adults.

### Medical expenditure and mental health among older adults

Aging degrades physical function, increasing older adults' vulnerability to diseases. Their deteriorating physical health and increased medical burden can also unfavorably influence their mental health (34). Research has demonstrated that among older adults with disabilities, negative emotions are significantly more serious than among those without disabilities (35). The deterioration of physical health inevitably leads to the increased utilization of medical services and additional medical expenditures (36). Moreover, chronic diseases or an increase in the types of chronic diseases significantly relate to high medical expenditures (37, 38). Health expenditures improve universal healthcare service coverage and life expectancy, but the association with the overall achievement of health outcomes is weak (39).

Some studies have noted that medical expenses are an important factor leading to patients' psychological stress. The amount of medical expenditure reflects changes in patients' physical health, utilization of medical services, and economic burden from medical treatment. On the one hand, medical insurance can significantly enhance older adults' use of medical services, thus improving physical and mental health and life satisfaction (40). On the other hand, most older adults in rural areas have no source of income, are vulnerable to the impacts of catastrophic medical expenditures, and can further experience poverty due to illness (41). Aging is an important element in households' catastrophic health payments in developing countries (12). Long-term medical treatments can cause patients to lose confidence in remaining healthy or alive, reduce their subjective well-being, and result in depression and anxiety, potentially even leading to suicide (42, 43). Hence, it is necessary to analyze whether medical expenditures significantly positively affected depression among rural older adults, specifically in terms of improving their physical health and reducing their medical burden.

Existing studies typically divide medical expenditures into “inpatient” and “outpatient” (44). First, theoretically, outpatient and inpatient medical treatments include different levels of medical behavior that correspond to different levels of disease severity. Second, the NRCMS provides a higher proportion of inpatient reimbursement to its participants. However, the data from four national health service surveys (45) reveal that the proportion of patients who should be hospitalized but are not increases significantly with age. Rural older adults also have economic constraints, with most choosing to receive outpatient medical services or purchase medicines not prescribed by a doctor, rarely utilizing inpatient medical services. Collectively, inpatient and non-inpatient medical expenditures include all medical service expenditures, and their impact on depression among older adults vary given different physical and family financial conditions. Therefore, this study further proposes the following hypotheses:

H2: Medical expenditures are associated with severe depression among rural older adults.

As medical expenditures are divided into two aspects, the following hypotheses are proposed:

H2a: Inpatient expenditures are associated with severe depression among rural older adults.

H2b: Non-inpatient expenditures are associated with severe depression among rural older adults.

### Intergenerational support and medical expenditure

Anderson's (46) behavioral model of health service use and its index system demonstrate that medical expenditures are affected by demographic characteristics such as income, gender, age, education, and various other factors including medical insurance, disease severity, and medical service pricing (47). Regarding older adults in particular, their participation in intra-household decision-making declines with age (48), and medical expenditures also relate to such factors as their social support and living arrangements (49). The theory of intergenerational support indicates that children's support impacts older adults' utilization of medical services and, consequently, their medical expenditures.

The effect of intergenerational support on medical expenditures is uncertain, with two opposing views, including substitution and complementary effects. Intergenerational support can improve older adults' health and shorten treatment times, partially replacing the use of medical services to reduce medical expenditures through a substitution relationship (50). Moreover, it can mitigate the demand for medical services among older adults by reducing access barriers and increasing inpatient medical expenditures through a supplementary relationship (51, 52). Nonetheless, the literature reveals that emotional comfort has no significant association with older adults' physical health (21) and no significant impact on their medical expenditures (53).

Generally, rural older adults are socioeconomically weak, and a high proportion refuse to seek medical treatment because they cannot afford it (54). A certain amount of economic support may improve their treatment rate, but more medical services will be replaced if their health improves under their children's care (53). Therefore, this study proposes the following hypotheses:

H3: Economic support has a positive impact on medical expenditures among rural older adults.

H4: Care-based support has a negative impact on medical expenditures among rural older adults.

Similarly, because medical expenditures include two aspects, the following assumptions are made:

H3a: Economic support has a positive impact on inpatient expenditures among rural older adults.

H3b: Economic support has a positive impact on non-inpatient expenditures among rural older adults.

H4a: Care-based support has a negative impact on inpatient expenditures among rural older adults.

H4b: Care-based support has a negative impact on non-inpatient expenditures among rural older adults.

The data for this study are sourced from the China Family Panel Studies (CFPS) data bank, during the years 2012, 2016, and 2018. The theory of intergenerational support and the behavioral model of health service use are drawn upon to explore the following objectives in the context of rural older adults: the impact of different types of intergenerational support and medical expenditures on depression; the impact of intergenerational support on medical expenditures; and the mediating role of medical expenditures in the relationship between intergenerational support and depression.

## Methods

### Sample selection

Data were obtained from the CFPS database, which is a long-term social survey of 25 provinces in China tracked every 2 years. Depression was measured by the Center for Epidemiologic Studies Depression Scale (CES-D) in 2012, 2016, and 2018 and by the Kessler Psychological Distress Scale (K6) in 2014. Thus, data from 2012, 2016, and 2018 were combined into a panel to facilitate comparisons and reduce any errors caused by different indicators. We selected rural older adults aged 60 and older as research subjects. After eliminating subjects who lacked key variables, we used a three-year balanced panel of 1,838 older adults who were surveyed in all three waves for a total of 5,514 observations.

### Variable description

#### Dependent variable

The dependent variable was depression assessed by CES-D score among rural older adults. There are a number of exhaustive diagnostic instruments for depression, such as the Composite International Diagnostic Interview, Diagnostic Interview Schedule and the CES-D (55). However, compared with instruments concerning clinical investigation, the CES-D created by Radloff is one of the most common instruments used for assessing depression in general populations globally (56–58); this tool measures the frequency of depressive events and ideas over the past week (59).

The original CES-D contains 20 items, of which 16 assess negative symptoms (e.g., “I felt lonely”) and four measure positive affect (e.g., “I was happy”) (60). Each item is rated on a four-point scale, with 0 representing “rarely or none (less than a day),” 1 indicating “unusual (1–2 days),” 2 indicating “often (3–4 days),” and 3 representing “most of the time (5–7 days).” The four positive items are reverse-scored. Total scores range from 0 to 60 points.

The CFPS used the 20-item CES-D to conduct a detailed survey on depression in 2012. Few respondents completed the survey in 2016 owing to its many questions: only 20% of adults answered all questions, and the other 80% answered the shorter version—only eight items. The same short version was adopted for all adults in 2018. According to the official CFPS database, an equi-percentile equating method was used in 2016 and 2018 to generate CES-D-20 score comparable to those in 2012. Therefore, the dependent variable is assessed by CES-D-20.

#### Independent variables

The explanatory variables were intergenerational support and medical expenditures (1) Based on the social support theory, scholars typically measure the aforementioned three types of intergenerational support: economic, care-based, and emotional (20). We constructed core variables according to items from the questionnaire module examining the relationship between children and parents: “Have your children provided economic support for you in the past 6 months?” “Have your children done housework or provided life care for you in the past 6 months?” and “How has the relationship between you and your children been in the past 6 months?” (2) Only one-third of the sample had inpatient medical expenditures. Based on existing research and according to the questionnaire—specifically, the items “How much was spent on medical treatment for hospitalization in the past year?” and “How much was the cost of disease, besides hospitalization, in the past year?”—medical expenditures were divided into inpatient and non-inpatient. Further, the total medical expenditures represent expenditure regardless of whether they were self-paid, and medical expenditures in the CFPS database include medical, treatment, or examination expenses, among others; preventive medical services are not included.

#### Covariates

According to existing research, older adults with depression are primarily women and characterized by their old age, low education, poor family income, and poor physical health. The number of children influences the intergenerational support older adults receive and can even directly impact their mood. The type of medical insurance is also expected to influence older adults who seek medical treatment. Therefore, this study controls for individual differences through demographic indicators (gender, age, and educational level), health status (self-rated health or the existence of chronic diseases), type of medical insurance, per capita family income, and number of children (61–64). Self-rated health in the CFPS questionnaire were measured by asking patients, “How would you rate your current health status?” with five possible responses: “very healthy,” “healthy,” “relatively healthy,” “general” or “unhealthy.” Regarding the type of medical insurance, the CFPS questionnaire has five categories with six options: “public medical insurance,” “urban employee medical insurance,” “urban residential medical insurance,” “NRCMS insurance,” “supplementary medical insurance,” and “none.” The description and assignment of variables are shown in Table 1.

**Table 1:** Variable definitions and statistical analysis.

**Variables**	**Description of variable settings**	**Mean (SD)**	**Percentage**	**References**
Depression (points)	The score of CES-D	22.00 (9.60)		(56)
Inpatient medical expenditure (yuan)	Inpatient medical expenditure	4,207.151 (14,085.21)		(14)
Non-inpatient medical expenditure (yuan)	Non-inpatient medical expenditure	1,417.643 (3,005.974)		(14)
Per capita family income (yuan)	Per capita family income	12,293.35 (85,624.09)		(65)
Number of children	Number of children	2.75 (1.48)		(66)
Age (years)	Age	68.75 (5.22)		(67)
Emotional support	The mean value of the emotional close degree with all children (very distant = 1; distant = 2; normal = 3; close = 4; very close = 5)	4.19 (0.71)		(68)
Economic support	Receiving children's economic support = 1 Not receiving = 0		83.33 16.67	(20)
Care support	Receiving children's economic support = 1 Not receiving = 0		75.61 24.39	(20)
Gender	Male = 1 Female = 0		53.84 46.16	(69)
Education level	Illiteracy = 1 Primary school = 2 junior high school or above = 3		60.50 26.34 13.16	(66, 69)
Type of medical insurance	None = 0 New rural cooperative medical insurance = 1 Urban resident medical insurance = 2 Urban employee medical insurance = 3 supplementary medical insurance = 4 public medical insurance = 5		4.36 88.67 1.93 3.36 0.30 1.38	(22)
Chronic diseases	Yes = 1 No = 0		27.04 72.96	(65)
Self-rated health level	Very healthy = 1 Health = 2 Relatively healthy = 3 General = 4 Unhealthy = 5		6.69 10.21 30.01 19.35 33.73	(69)

The sample's average age was 68.75 years. In terms of gender, 53.84% were men, and 46.16% were women. Regarding education, 86.84% had an educational level of primary school or below, and those with junior high school education and above accounted for only about 13.16%; overall, the respondents' educational level was low. In terms of depression, the mean CES-D score was 22, meaning that the majority of older adults experienced depressive symptoms. Regarding intergenerational support from children, 83.33% received economic support, 75.61% received care-based support, and the mean value for emotional support was 4.19, indicating generally close relationships between older adults and their children. Regarding medical expenditures, the mean values of inpatient and non-inpatient costs were 4,207.151 yuan and 1,417.643 yuan, respectively. In terms of per capita family income, the average value was 12,293.35 yuan. In regard to the type of medical insurance, the vast majority (95.64%) had medical insurance, and most (88.67%) had NRCMS insurance. Finally, regarding self-rated health, 6.69% reported being very healthy, 10.21% reported being healthy, nearly half reported being relatively healthy, and 33.73% reported being unhealthy.

### Statistical analysis

The panel data model can address the endogeneity caused by unobservable individual heterogeneity and correctly interpret the relationship between variables; we use this approach to investigate the effect of intergenerational support and medical expenditures on depression among the older adults. We estimated the following model using three-wave panel data:


$$\begin{array}{l} D_{it}\;=\;\alpha_{0}+\beta_{1}S_{it}+\lambda_{1}Control_{it}+\lambda_{2}Year_{it} \end{array}$$

(1) $$\begin{array}{l} +\lambda_{3}Region_{it}+\varepsilon_{it} \end{array}$$

$$\begin{array}{l} D_{it}\;=\;\alpha_{0}+\beta_{2}IME_{it}+\beta_{3}NME_{it}+\lambda_{4}Control_{it} \end{array}$$

(2) $$\begin{array}{l} +\lambda_{5}Year+\lambda_{6}Region+\varepsilon_{it} \end{array}$$

$$\begin{array}{l} IME_{it}\;=\;\alpha_{0}+\beta_{4}S_{it}+\lambda_{7}Control_{it}+\lambda_{8}Year \end{array}$$

(3) $$\begin{array}{l} +\lambda_{9}Region+\varepsilon_{it} \end{array}$$

$$\begin{array}{l} NME_{it}\;=\;\alpha_{0}+\beta_{5}S_{it}+\lambda_{10}Control_{it}+\lambda_{11}Year \end{array}$$

(4) $$\begin{array}{l} +\lambda_{12}Region+\varepsilon_{it} \end{array}$$

$$\begin{array}{l} D_{it}\;=\;\alpha_{0}+\beta_{6}S_{it}+\beta_{7}IME_{it}+\beta_{8}NME_{it} \end{array}$$

(5) $$\begin{array}{l} +\lambda_{13}Control_{it}+\lambda_{14}Year+\lambda_{15}Region+\varepsilon_{it} \end{array}$$

where *D*_*it*_represents depression among older adults that measured by the CES-D; *S*_*i*_ is the key independent variable representing intergenerational support, which includes economic, care-based, and emotional support;*IME*_*it*_ represents inpatient medical expenditures, and*NME*_*it*_ represents non-inpatient medical expenditures;*Control*_*it*_ represents all of the control variables, including demographic indicators (gender, age, and educational level), health status (self-rated health changes and existence of chronic diseases), type of medical insurance, per capita family income, and the respondent's number of children. Additionally, *Year* and *Region* are virtual control variables; ε_*it*_ represents the random error term of the mixed difference between respondents and time; *i*, *t* represents data from the *i*^th^ older adult respondent in year *t*.

In general, panel data models include random effects (RE) models and fixed effects (FE) models, and the Hausman test is used to determine which model to apply. The data were analyzed using Stata/SE version 15.0 for Windows (StataCorp, College Station, TX, USA).

## Results

### Statistical inference

Table 2 indicates the results of analysis of variance concerning depression and medical expenditures in the context of whether or not older adults received intergenerational support. In terms of depression, the mean CES-D score among older adults who received children's economic support was 9.77 points higher than the mean score of those who did not receive such support. Further, the mean CES-D score among older adults who received care-based support was 11.77 points higher than among their counterparts. Regarding emotional support, the mean CES-D score of recipients was lower than that of older adults who did not receive such support. In terms of medical expenditures, the inpatient costs of those who received economic or care-based support were higher than the costs of those who did not receive such support. However, there were no significant differences in inpatient medical expenditures based on the presence or absence of emotional support. Moreover, non-inpatient medical expenditures among older adults who received intergenerational support were not significantly different from those who did not receive intergenerational support. Thus, intergenerational support may have an impact on inpatient medical expenditures and depression.

**Table 2:** Analysis of variance results of depression and medical expenditures among the rural elderly in the context of whether or not older adults received intergenerational support.

**Variables**	**Sample size**	**Mean**	**Sample size**	**Mean**	**Difference**
	**Not receiving children's economic support**		**Receiving children's economic support**		**Difference test**
Ln (inpatient medical expenditure)	842	0.79	1,469	5.11	4.32^***^
Ln (non-inpatient medical expenditure)	743	5.32	4,018	5.29	0.03
CES-D score	842	14.55	4,210	24.32	9.77^***^
	**Not receiving children's care support**		**Receiving children's care support**		**Difference test**
Ln (inpatient medical expenditure)	1,232	0.79	1,079	6.67	5.88^***^
Ln (non-inpatient medical expenditure)	1,085	5.41	3,676	5.26	−0.15
CES-D score	1,232	14.25	3,820	25.42	11.17^***^
	**Not receiving children's emotional support**		**Receiving children's emotional support**		**Difference test**
Ln (inpatient medical expenditure)	40	3.31	2,634	3.28	−0.03
Ln (non-inpatient medical expenditure)	69	5.06	5,086	5.29	0.23
CES-D score	78	27.40	5,436	22.33	−5.07^***^

^*^, ^**^,

*** represent the significance level of 10, 5, and 1%, respectively.

### Impact of intergenerational support and medical expenditure on depression

Table 3 presents the impacts of not only intergenerational support and medical expenditures on depression among rural older adults but also of intergenerational support on medical expenditures. The findings of the Hausman test support the fixed-effect model in the benchmark regression (prob > chi^2^ = 0.001); the virtual variables “year” and “region” were controlled for. Model 1 presents the regression results of the impact of different types of intergenerational support on depression among rural older adults. The results reveal that intergenerational support was significantly associated with CES-D score, and older adults who received economic support (*β* = −0.854, *p* < 0.05) or had closer intergenerational relationships (*β* = −0.890, *p* < 0.01) were likely to experience lower CES-D score; thus, H1a and H1c were supported. In contrast, receiving care-based support was significantly positively associated with CES-D score at a 1% significance level (*β* = 2.003, *p* < 0.01); thus, H1b was disproved. Additionally, the number of children was negatively associated with CES-D score among older adults at a 5% significance level, implying that the concept of “more children, more happiness” is still prevalent in rural areas.

**Table 3:** Regression analysis for intergenerational support and medical expenditures on depression among the rural older adults.

**Variables**	**Model 1**	**Model 2**	**Model 3**	**Model 4**	**Model 5**
	**CES-D score**	**CES-D score**	**Inpatient**	**Non-inpatient**	**CES-D score**
Economic support (ref: not receiving)	−0.854^**^		0.564^**^	−0.074	−0.542
	(0.398)		(0.280)	(0.187)	(0.853)
Care support (ref: not receiving)	2.003^***^		0.665^**^	−0.453^*^	2.555^***^
	(0.498)		(0.338)	(0.238)	(1.058)
Emotional support	−0.890^***^		−0.004	0.051	−1.191^***^
	(0.173)		(0.149)	(0.079)	(0.438)
Ln (inpatient medical expenditure)		0.278			−0.168
		(0.255)			(0.284)
Ln (non-inpatient medical expenditure)		0.304^***^			0.365^***^
		(0.101)			(0.107)
Number of children	−0.492^**^	0.368	−0.354	0.080	0.418
	(0.266)	(0.372)	(0.227)	(0.120)	(0.651)
Chronic diseases (ref: no)	0.806^***^	1.011^*^	0.273	0.761^***^	1.119^*^
	(0.259)	(0.564)	(0.197)	(0.118)	(0.582)
Self-rated health level = 2 (ref: very healthy)	0.307	−0.390	−0.917	0.332	0.601^**^
	(0.533)	(1.527)	(0.596)	(0.237)	(0.284)
Self-rated health level = 3 (ref: very healthy)	1.034^**^	0.619	0.616^***^	0.692^***^	0.803
	(0.485)	(1.458)	(0.554)	(0.217)	(1.267)
Self-rated health level = 4 (ref: very healthy)	1.402^***^	−0.855	0.191	0.973^***^	1.285
	(0.518)	(1.496)	(0.560)	(0.233)	(0.887)
Self-rated health level = 5 (ref: very healthy)	3.498^***^	2.035	0.441^***^	1.486^***^	2.037^**^
	(0.523)	(1.454)	(0.126)	(0.236)	(0.686)
Type of medical insurance = 1 (ref: none)	−0.691	2.841	−0.808	0.522^**^	3.289
	(0.549)	(1.934)	(0.705)	(0.248)	(2.115)
Type of medical insurance = 2 (ref: none)	−1.554	1.927	0.085	0.467	0.763
	(1.074)	(3.221)	(1.162)	(0.493)	(3.378)
Type of medical insurance = 3 (ref: none)	−2.391^**^	−0.983	0.365	1.389^***^	−1.750
	(1.129)	(2.538)	(0.955)	(0.505)	(2.733)
Type of medical insurance = 4 (ref: none)	−1.419	−3.622	−1.112	0.973	−0.979
	(1.877)	(4.470)	(1.571)	(0.882)	(5.007)
Type of medical insurance = 5 (ref: none)	−1.579	0.341	−0.726	0.710	−1.481
	(1.316)	(2.738)	(1.083)	0.590	(3.021)
Age	0.352	−0.260	0.138	0.141	−0.346
	(0.260)	(0.609)	(0.216)	(0.120)	(0.599)
Gender (ref: female)	−1.075	3.947	−1.553	4.951^**^	2.371
	(4.892)	(8.493)	(3.093)	(2.122)	(8.434)
Education level 2 (ref: Education level 1)	−1.446	1.253	0.996	0.397	1.309
	(1.341)	(2.764)	(0.950)	(0.616)	(2.897)
Education level 3 (ref: Education level 1)	2.572	2.363	0.016	−0.012	1.353
	(3.117)	(3.011)	(1.235)	(1.353)	(2.214)
Ln (per capita family income)	−0.385^***^	−0.847^***^	0.031	0.165^***^	−0.695^**^
	(0.130)	(0.270)	(0.095)	(0.060)	(0.287)
Constant	−0.596	30.419	−6.295	−9.903	39.199
	(17.307)	(40.286)	(14.396)	(7.938)	(40.045)
Regional fixed effect	Yes	Yes	Yes	Yes	Yes
Time fixed effect	Yes	Yes	Yes	Yes	Yes
R-squared	0.546	0.631	0.825	0.055	0.628

Standard errors in parentheses,

*, **, *** represent the significance level of 10%, 5%, and 1%, respectively.

Model 2 presents the regression results of the impacts of medical expenditures on depression among rural older adults, in which non-inpatient medical expenditures were significantly positively associated with CES-D score (*β* = 0.304, *p* < 0.01). As inpatient medical expenditures did not produce a significant influence (*β* = −0.270, *p* > 0.1), H2b was proven, but H2a was not verified. Further, rural older adults may report higher CES-D score when experiencing chronic diseases or when their self-rated health is poor, as demonstrated by the results from Models 1 and 2. Moreover, per capita family income was significantly negatively associated with CES-D score at the 1% significance level. However, age, gender, education, and the type of medical insurance were not significantly related to CES-D score.

Models 3 and 4 denote the regression results of the impact of intergenerational support on medical expenditures among rural older adults, and the explained variables are inpatient and non-inpatient medical expenditures. Economic support has a positive impact on inpatient medical expenditures (*β* = 0.564, *p* < 0.05); hence, H3a was verified. However, an insignificant influence on non-inpatient medical expenditures existed; thus, H3b was not supported. This is possibly because Chinese older adults, especially those living in rural areas, have a stronger traditional notion of saving their money for untimely needs rather than investing in health in daily life (70). Care-related support significantly affected medical expenditures, as respondents who received care-based support had more inpatient medical expenditures (*β* = 0.665, *p* < 0.05) and lower non-inpatient medical expenditures (*β* = −0.453, *p* < 0.1), disproving H4a and proving H4b. However, emotional support had no significant impact on medical expenditures.

In contrast, non-inpatient medical expenditures were significantly positively associated with worse self-rated health, existence of chronic disease, type of medical insurance, per capita family income, and gender. Older adults diagnosed with chronic diseases in the past 6 months had higher non-inpatient medical expenditures (*β* = 0.761, *p* < 0.01). Older adults who participated in medical insurance programs—such as NRCMS (*β* = 0.522, *p* < 0.05) or urban employees' basic medical insurance (*β* = 1.389, *p* < 0.01)—were more likely to have more non-inpatient medical expenditures. Additionally, per capita family income was positively correlated with the use of health services and non-inpatient medical expenditures by decreasing the medical burden. Finally, gender was associated with non-inpatient medical expenditures; men were more likely to have higher such expenditures. However, the number of children and age were not significantly correlated with non-inpatient medical expenditures.

Model 5 displays the regression result of adding all independent variables. Economic support had a negative influence on CES-D score in older adults after adding medical expenditures as a variable, but it was not significant. Care-based support was positively associated with higher CES-D score in older adults (*β* = 2.555, *p* < 0.01), and those with more emotional support reported lower CES-D score (*β* = −1.191, *p* < 0.01), consistent with the results of Model 1. Non-inpatient medical expenditures were significantly and positively correlated with higher CES-D score (*β* = 0.365, *p* < 0.01), consistent with the results of Model 2. Moreover, the mediating roles of inpatient and non-inpatient medical expenditures in the relationship between intergenerational support and depression were examined and reported in the Supplementary Materials, which indicated that the mediating role of medical expenditures between intergenerational support and depression was not significant.

### Robustness test based on propensity score matching

The benchmark regression analysis indicated that the effects of care-based and emotional support and non-inpatient medical expenditures on depression were significant, while the impact of economic and care-based support on medical expenditures was significant. However, individual differences in factors can determine whether older adults are supported by their children or incur medical expenditures. Some respondents did not report medical expenditures, causing the problem of a missing variable; the covariates were associated not only with depression but also potentially with intergenerational support, causing endogeneity problems. Therefore, the propensity score matching (PSM) method was used to conduct counterfactual analysis to reduce sample selection bias and resolve the issues of missing data and endogeneity, thus improving the regression results' reliability (66, 71).

The core variables that significantly impacted the outcome variables were regarded as the treatment variables. The *k*-nearest neighbor-matching method (*n* = 4) was used to test the average treatment effect on the treated (ATT) of intergenerational support and medical expenditures on CES-D score as well as the ATT of intergenerational support on medical expenditures by comparing the two samples, which differ only in terms of the key processing variables and have similar resource endowment characteristics. As Table 4 indicates, the net effect of care-based support (*t* = 2.02, *p* < 0.05), emotional support (*t* = −2.37, *p* < 0.05), and non-inpatient medical expenditures (*t* = 3.50, *p* < 0.01) on CES-D score was significant, indicating that the former regression analysis results were robust and disproving H1b. Furthermore, H1c and H2b endorsed the PSM test.

**Table 4:** The Propensity score matching (PSM) test for the treatment variable.

**Treatment variables**	**Sample**	**ATT**	**S.E**.	**T**
	**CES-D score**			
Economic support	Unmatched	0.04	0.54	0.08
	Matched	−0.23	0.67	−0.34
Care support	Unmatched	1.91	0.69	2.76
	Matched	1.72	0.85	2.02^**^
Emotional support	Unmatched	−6.70	1.17	−5.71
	Matched	−4.86	2.06	−2.37^**^
Non-inpatient medical expenditure	Unmatched	1.64	0.33	4.91
	Matched	1.76	0.50	3.50^***^
	**Inpatient medical expenditure**			
Economic support	Unmatched	0.22	0.15	1.50
	Matched	−0.09	0.18	−0.49
Care support	Unmatched	0.55	0.19	2.92
	Matched	0.35	0.24	1.47
	**Non-inpatient medical expenditure**			
Economic support	Unmatched	0.16	0.15	1.05
	Matched	0.12	0.18	0.63
Care support	Unmatched	−0.38	0.20	−1.92
	Matched	−0.52	0.24	−2.16^**^

^*^,

**, *** represent the significance level of 10, 5, and 1%, respectively.

Regarding inpatient medical expenditures, the ATT of economic support and care-based support were lower than that of the regression coefficient in the fixed-effects Model 3, and the *t*-test was not significant. Thus, economic and care-based support had an insignificant net effect on inpatient medical expenditures after matching. Regarding the effect of intergenerational support on non-inpatient medical expenditures, the matched ATT of care support (ATT = −0.52) was close to the regression coefficient in Model 4 (*β* = −0.453). However, economic support had an insignificant effect on non-inpatient medical expenditures. Thus, care-based support had the potential to reduce non-inpatient medical expenditures but report higher CES-D scores; emotional support had a significantly negative net effect on CES-D score; and non-inpatient medical expenditures had a positive net effect on CES-D score. However, the effects of economic support on CES-D score and medical expenditures did not pass the PSM test, nor did care support's effect on inpatient medical expenditures.

### Heterogeneity in impact of intergenerational support on medical expenditure and depression

The need for intergenerational support and medical services varies among older adults with different physical conditions and ages. First, the sample was divided to delineate whether the individual had experienced a chronic disease in the last half of the year. Second, the sample was divided into a “young” group, or those under age 75, and a “very old” group, or those aged 75 or older, according to the World Health Organization's age division criteria. The impact of intergenerational support and medical expenditures on depression among older adults and the impact of intergenerational support on medical expenditures were analyzed using a grouped ordinary least-squares regression in the different groups, including cases of chronic diseases (or none) as well as different ages. The Hausman test is used to decide the regression model either with or without fixed effects. Tables 5, 6 present the results.

**Table 5:** Sub-samples regression results regarding the effects of intergenerational support and medical expenditures on depression among the rural older adults.

**Variables**	**CES-D score**			
	**Chronic** = **0**	**Chronic** = **1**	**Age**<**75**	**Age**≥**75**
	***N*** = **4,023**	***N*** = **1,491**	***N*** = **4,711**	***N*** = **803**
Ln (inpatient medical expenditure)	−0.094	0.114	−0.138	−0.025
	(0.257)	(0.314)	(0.318)	(0.454)
Ln (non-inpatient medical expenditure)	0.282^***^	0.215	0.326^***^	0.151
	(0.078)	(0.156)	(0.126)	(0.175)
Economic support (ref: not receiving)	−0.473	−0.529	−0.625	0.083
	(0.500)	(1.114)	(0.981)	(2.193)
Care support (ref: not receiving)	1.204^*^	2.507^*^	2.877^**^	−2.494
	(0.642)	(1.357)	(1.224)	(2.980)
Emotional support	−1.492^***^	−1.372^***^	−2.049^***^	−3.345^***^
	(0.301)	(0.460)	(0.475)	(0.764)
Covariates	Controlled	Controlled	Controlled	Controlled
Regional fixed effect	No	No	Yes	No
Time fixed effect	No	No	Yes	No
R-squared	0.507	0.441	0.619	0.622

Standard errors in parentheses,

*, **, *** represent the significance level of 10, 5, and 1%, respectively.

**Table 6:** Sub-samples regression results regarding the effect of intergenerational support on the medical expenditures of the rural older adults.

**Variables**	**Chronic** = **0** ***N*** = **4,023**		**Chronic** = **1** ***N*** = **1,491**		**Age**<**75** ***N*** = **4,711**		**Age**≥**75** ***N*** = **803**	
	**Inpatient**	**Non-inpatient**	**Inpatient**	**Non-inpatient**	**Inpatient**	**Non-inpatient**	**Inpatient**	**Non-inpatient**
Economic support (ref: not receiving)	0.321^**^	−0.032	−0.506^*^	0.357	0.598^*^	−0.025	0.692	−0.505
	(0.140)	(0.192)	(0.291)	(0.313)	(0.329)	(0.206)	(0.520)	(0.816)
Care support (ref: not receiving)	0.189	−0.604^**^	1.460^***^	−0.309	0.939^**^	−0.473^*^	0.765	−1.185
	(0.177)	(0.247)	(0.344)	(0.384)	(0.393)	(0.263)	(0.675)	(1.124)
Emotional support	−0.018	0.062	−0.034	−0.003	0.093	0.114	0.250	0.016
	(0.084)	(0.073)	(0.128)	(0.095)	(0.166)	(0.088)	(0.188)	(0.158)
Covariates	Controlled	Controlled	Controlled	Controlled	Controlled	Controlled	Controlled	Controlled
Regional fixed effect	No	No	No	No	Yes	Yes	No	No
Time fixed effect	No	No	No	No	Yes	Yes	No	No
R-squared	0.750	0.149	0.715	0.092	0.819	0.052	0.828	0.270

Standard errors in parentheses,

*, **, *** represent the significance level of 10, 5, and 1%, respectively.

The sample heterogeneity in Table 5 demonstrates that care-based support positively correlated with CES-D score among young older adults (*β* = 2.877, *p* < 0.05) and negatively correlated with CES-D scores in the very old (*β* = −2.494, *p* > 0.1), although the association was not significant. Emotional support had negative influence on CES-D score in the full sample at the 1% level, and the coefficient was higher for the very old (*β* = −3.345). Similarly, the regression results regarding medical expenditures' impact on CES-D score revealed that non-inpatient medical expenditures were positively associated with CES-D score among the older adults. Moreover, those with no chronic diseases (*β* = 0.282, *p* < 0.01) and the young group (*β* = 0.326, *p* < 0.01) were more sensitive to medical expenditures.

The results in Table 6 indicate that emotional support had no significant impact on medical expenditures, while economic support positively impacted inpatient medical expenditures for those with no chronic diseases (*β* = 0.321, *p* < 0.05) and negatively impacted inpatient medical expenditures for those with chronic diseases (*β* = −0.506, *p* < 0.1). Care-based support had a positive influence on inpatient medical expenditures for those with chronic diseases (*β* = 1.460, *p* < 0.01) and the young group (*β* = 0.939, *p* < 0.01) and a negative influence on non-inpatient medical expenditures for those without chronic diseases (*β* = −0.604, *p* < 0.05) and the young group (*β* = −0.473, *p* < 0.1).

In conclusion, according to the regression analysis and robustness test results, there was heterogeneity in the impact of different types of intergenerational support on depression among rural older adults. All of the samples indicated that economic support was negatively correlated with CES-D score, but this was insignificant; thus, H1a could not be verified. Care-based support was significantly associated with CES-D score. Specifically, it had a significant and positive impact on CES-D score among the young group and a negative but insignificant correlation with CES-D score among the very old; hence, H1b was refuted. In contrast, emotional support significantly and negatively impacted CES-D score, verifying H1c. While medical expenditures—which represent both physical health and medical burden—had an adverse effect on CES-D score in older adults, only non-inpatient medical expenditures were significant; therefore, while H2a could not be verified, H2b was proved.

The regression results regarding the impact of intergenerational support on medical expenditures demonstrate that those with economic support may have more medical expenditures, but the net effect on inpatient medical expenditures did not pass the PSM test. Thus, H3a could not be verified, but H3b was verified. The results of the sub-sample regression show that economic support was positively associated with inpatient medical expenditures among older adults without chronic diseases and negatively associated with such expenditures among older adults with chronic diseases. Older adults with care-based support generated more inpatient medical expenditures and lower non-inpatient medical expenditures; however, non-inpatient medical expenditures' mediating role in care-based support's influence on depression was insignificant. Additionally, the robustness test results shown in the Supplementary Materials were obtained by replacing the explanatory variables, which revealed that the older adults who received higher-frequency care-based support reported higher inpatient medical expenditures; thus, H4b was supported, while H4a was not. The sub-sample regression results demonstrated that receiving care-based support was positively correlated with the inpatient medical expenditures of older adults with chronic diseases and the younger group. In contrast, it was negatively correlated to non-inpatient medical expenditures among older adults without chronic diseases and those younger than 75.

In terms of covariates, respondents with chronic diseases or worse self-rated health were more likely to report higher CES-D scores, and higher per capita family income was correlated with lower CES-D scores in this population. Moreover, the family's economic level was also positively associated with non-inpatient medical expenditures for the younger group.

## Discussion

Based on the CFPS balanced panel data from 2012, 2016, and 2018, the regression and robustness test results demonstrated the existence of heterogeneity in the impact of different types of intergenerational support on depression among rural older adults. Further, the impact of medical expenditures on depression and the impact of intergenerational support on medical expenditures differed among the indicators. The specific analysis is as follows.

As intergenerational support was partially associated with depression among rural older adults, H1 was partially proven. The regression results indicated that older adults experienced lower CES-D scores with economic support, but this was not significant; hence, H1a could not be verified. Most studies generally state that economic support acts as a buffer against the effects of life costs on depression among older adults (72). However, consistent with our conclusion, some scholars posit that economic support has no significant effect on older adults' physical and mental health (73). Based on the 2014 Chinese General Social Survey database, He et al. (21) classified all children's economic support in that year into three levels—low, medium, and high—according to the following ranges: < 1,000 yuan, 1,000 to 5,000 yuan, and more than 5000 yuan, respectively. For rural older adults, medium and high economic support was associated with significantly fewer depressive symptoms, but low economic support was insignificant in this regard. This study's statistical results showed that in the past 6 months, 921 older adults received low economic support (< 500 yuan) in 2016 and 774 in 2018; 508 and 591 received medium economic support (500 to 2,500 yuan) in 2016 and 2018, respectively; and 409 and 473 received high economic support (> 2,500 yuan) in 2016 and 2018, respectively. Half of the older adults received low economic support, or < 83 yuan per month. Among these, 842, 814, and 628 older adults did not receive economic support from their children in 2012, 2016, and 2018, respectively; thus, the economic support provided by children in rural China is generally limited (74). As it is difficult for older adults to reduce their life stress, this factor was insignificant with regard to lower CES-D scores.

As care-based support significantly and positively affected depression among rural older adults, H1b was disproved. Some scholars believe that family care can increase the interactions between older adults and their family members, thus alleviating depression and enhancing their life satisfaction (75), which contradicts this study. Specifically, most of the present respondents were younger than 75 (*n* = 4,711) or did not have chronic diseases (*n* = 4,023). First, this predisposes them to “excessive” care. Younger older adults have some self-care ability, and children's life care can easily interfere in their lives, producing psychological stress. It may also undermine their evaluations of self-perceived health and self-utility or lead to intergenerational conflict, thus producing severe depression (53, 76). Early studies based on older female adults also indicate that family care affects their privacy to some extent, consequently deteriorating their mental health (77). Second, as rural families commonly have a low socioeconomic status, most young migrants move to other areas to work. The opportunity cost incurred by children remaining in the countryside causes psychological stress for older adults, and long-term care generates guilt (78). Third, based on intergenerational reciprocity theory, special residential arrangements for older adults in rural China have led to lower expectations for nursing care from non-coresidential children. As children in rural areas become adults, older adults primarily live with their sons (79, 80), and any other children are relatively estranged, especially after a daughter is married. Parents invest heavily in the child they live with, thus expecting greater returns with regard to nursing care. As a result, care-based support from non-coresidential children, especially daughters, places a psychological burden on older adults.

The regression results revealed that compared with economic and care-based support, emotional support and close relationships had a negative impact on CES-D score among older adults, and this effect was stronger for those with chronic diseases or who were older than 75, verifying H1c. Intergenerational relationship intimacy potentially alleviated depression in rural older adults (25, 26). However, in other surveys, children in rural areas focused more on economically supporting their parents, followed by care-based support, and were least likely to offer emotional support (81). Family members should be encouraged to pay more attention to older adults' emotional needs and their intimate relationship to relieve their loneliness, isolation, and depression.

The impact of medical expenditures on depression among rural older adults was partially significant, and H2 was partly proven, with the regression results indicating that non-inpatient medical expenditures significantly and positively influenced CES-D score. Hence, H2a was verified. Existing studies have noted that poor physical health is detrimental to mental health (63, 82). However, older adults are vulnerable to disease shocks, and those in rural areas have multiple psychological burdens, such as life constraints, poor physical health, and high medical expenditures (83). Although some studies have suggested that higher medical expenditures are beneficial for improving older adults' life satisfaction, the family's economic status is also significantly associated with depression among older adults (84). High medical expenditures in rural areas can create anxiety among older adults, as they can perceive themselves as a burden to the family, leading to more serious phenomena such as depression or even suicide (85). Nevertheless, inpatient medical expenditures had no significant impact on CES-D score; thus, H2b could not be verified. The proportion of inpatient medical reimbursements under the NRCMS policy was high, while the proportion of inpatient services used by rural older adults was low owing to the reimbursement threshold. Thus, the amount of non-inpatient medical expenditures, as the primary medical expenditures, had a stronger positive impact on higher CES-D scores among older adults.

Economic support was partially associated with rural older adults' medical expenditures, partially verifying H3. The regression results revealed that economic support positively influenced inpatient medical expenditures for those with no chronic diseases, indicating that economic support was beneficial in reducing their economic burden and increasing medical expenditures (53). However, economic support's effect on medical expenditures did not pass the PSM test because economic support in rural areas is not sufficient. The robustness test conducted by replacing explanatory indicators demonstrated that increasing economic support had a positive impact on non-inpatient medical expenditures. In Chen et al.'s study (86) based on the CHARLS database, economic support was limited, which significantly affected middle-income older adults' physical health and weakly affected high- and low-income older adults. Hence, the effects of economic support were correlated with the amount of support and older adults' income.

Second, care-based support was partially associated with medical expenditures among rural older adults, partially verifying H4. The regression results showed that care-based support significantly and negatively affected older adults' non-inpatient medical expenditures, verifying H4b. This is consistent with existing studies wherein care-based support had an alternative effect on healthcare services by improving older adults' health through the provision of life care (53). However, care-based services have a limited effect on physical health (21); this explains the non-significant impact on non-inpatient medical expenditures among older adults with chronic diseases or who were older than 75. In the case of sudden illness or severe diseases, care services can reduce the barriers to medical treatment (51, 52), which exerts a complementary effect on medical services and elevates inpatient medical expenditures. The sub-sample regression indicated that the impact was significant for older adults with chronic diseases, but the net effect did not pass the PSM test. Hence, H4a was disproved.

Moreover, intergenerational support directly affected older adults' mood, and the mediating role of medical expenditures between intergenerational support and depression was not significant. Therefore, as it is difficult to alleviate depression in older adults by reducing medical expenditures through intergenerational support from adult children, it is necessary to alleviate their medical burden *via* formal support.

Compared with previous studies, this study is novel in certain aspects. First, two factors—intergenerational support and medical expenditures—were considered to have the greatest influence on depression among rural older adults because social nursing and medical security in rural China still need improvement. Second, this work comprehensively explores the effects of intergenerational support in three dimensions: economic, care, and emotional support. The results revealed that the different types of intergenerational support had varying correlations with depression among rural older adults. Compared with economic and care-based support, emotional support had a significantly negative influence on depression among older adults, while excessive care support was positively associated with severe depression and low economic support had no significant effect. Third, the group differences in previous studies were primarily analyzed given various categories of gender or age, among other factors. In addition to considering different age groups, this study conducted a sub-sample regression given different health levels, specifically, whether older adults were diagnosed with chronic diseases in the past 6 months. This led to an analysis of differences in the impact of intergenerational support and medical expenditures on depression as well as the impact of intergenerational support on medical expenditures among older adults with different physical conditions. This work demonstrated that among respondents with care support, higher CES-D scores were reported for younger older adults, and care support partially replaced the use of non-inpatient medical services for those without chronic diseases or who were younger than 75.

This study has several limitations. First, we focused on rural older adults and primarily considered intergenerational support from adult children, which did not include intergenerational mutual support or support provided by grandchildren or spouses. Second, the study did not explore the interactions among different types of intergenerational support. Third, the examination was limited to the CFPS database, which had samples that were mostly younger than 75 years, resulting in a younger sample. Finally, while the fixed- effects model and PSM could solve the endogeneity problem to some extent, this problem cannot be completely resolved, and potential reverse causality should be considered in further studies.

## Conclusions and implications

First, heterogeneity was observed in the impact of intergenerational support on depression among China's rural older adults in different dimensions. Emotional support had a negative impact on depression, and excessive care support had a positive impact on depression, except among older adults aged above 75. In contrast, low-level economic support had no significant impact on depression. Therefore, more attention should be paid to the mental health problems of older adults in rural areas, especially among those with chronic diseases. It is necessary to advocate for and motivate intergenerational intimacy and emotional support in contemporary society. Further, the stress from intergenerational support should be reduced by formulating incentives, such as financial allowances and nursing training, which encourage children to provide high-level economic support and effective care to older adults. This is especially essential for older adults in poor physical health or of advanced age, thus relieving depression.

Second, medical expenditures are an economic burden for rural older adults, among which there is a high proportion of individuals who should be hospitalized but are not. Therefore, non-inpatient medical expenditures had a greater emotional impact as the primary type of medical expenditure. Further, higher non-inpatient medical expenditures significantly aggravate depression. It is necessary to focus on chronic disease care to improve rural medical systems. This will reduce the problem of the difficulty and high cost of obtaining medical services among rural older adults. Moreover, a medical insurance reimbursement policy for chronic diseases should be implemented for rural residents. The reimbursement application process should also be simplified to reduce medical expenditures and alleviate the associated psychological stress.

Finally, the impact of intergenerational support on medical expenditures can be divided into two levels. First, the increase in economic support, which is accompanied by an increase in older adults' non-inpatient medical expenditures, has a supplementary effect. Second, receiving care-based support would produce lower non-inpatient medical expenditures as a substitution effect. The family security system should be fully implemented to capitalize on the traditional filial piety culture, and intergenerational support is encouraged to improve older adults' ability to pay for medical services. However, the fact that the mediating role of medical expenditures in the relationship between intergenerational support and depression was not significant implies that it is difficult to alleviate depression among older adults by reducing medical expenditures through children's support. Thus, subsidies for older adults, as formal support, should also be improved for low-income older adults, especially in rural areas and if their children's economic support is limited, to alleviate depression caused by life pressures and medical burdens.

## Data availability statement

Publicly available datasets were analyzed in this study. This data can be found at: http://www.isss.pku.edu.cn/cfps/.

## Author contributions

CL contributed to the study conception and research design. QH conducted the statistical analysis and contributed to the drafting of the manuscript. JH, ZH, and HY provided critical feedback and contributed to the discussion. CL and QH edited the manuscript for language. All authors contributed to the revision of the manuscript and have read and approved the submitted version.

## Funding

This work was supported by the National Social Science Fund of China (Grant No. 18XRK001).

## Conflict of interest

The authors declare that the research was conducted in the absence of any commercial or financial relationships that could be construed as a potential conflict of interest.

## Publisher's note

All claims expressed in this article are solely those of the authors and do not necessarily represent those of their affiliated organizations, or those of the publisher, the editors and the reviewers. Any product that may be evaluated in this article, or claim that may be made by its manufacturer, is not guaranteed or endorsed by the publisher.
