# Supplementary material for: Impact of intergenerational support and medical expenditures on depression: Evidence from rural older adults in China
Source: Front Public Health. 2022 Jul 22;10:840864. doi: 10.3389/fpubh.2022.840864 (PMC9354659; doi:10.3389/fpubh.2022.840864)
Supplement: Supplementary file 1 [file Table_1.docx]

Supplementary Materials

**Supplementary Table 1** Regression analysis regarding medical expenditures’ mediating role in the relationship between intergenerational support and depression

| Variables | Model 1 | Model 2 | Model 3 | Model 4 | Model 5 | Model 6 |
| --- | --- | --- | --- | --- | --- | --- |
|  | Depression | Depression | Inpatient | Non-inpatient | Depression | Depression |
| Economic support (ref: not receiving) | -0.854^**^ |  | 0.564^**^ | -0.074 | -1.337^*^ | -0.579 |
|  | (0.398) |  | (0.280) | (0.187) | (0.770) | (0.416) |
| Care support (ref: not receiving) | 2.003^***^ |  | 0.665^**^ | -0.453^*^ | 1.833^**^ | 2.174^***^ |
|  | (0.498) |  | (0.338) | (0.238) | (0.930) | (0.533) |
| Emotional support | -0.890^***^ |  | -0.004 | 0.051 | -1.131^***^ | -0.737^***^ |
|  | (0.173) |  | (0.149) | (0.079) | (0.409) | (0.176) |
| Ln (inpatient medical expenditure) |  | 0.278 |  |  | 0.046 |  |
|  |  | (0.255) |  |  | (0.112) |  |
| Ln (Non-inpatient medical expenditure) |  | 0.304^***^ |  |  |  | 0.128^***^ |
|  |  | (0.101) |  |  |  | (0.043) |
| Number of children | -0.492^**^ | 0.368 | -0.354 | 0.080 | 0.116 | -0.305 |
|  | (0.266) | (0.372) | (0.227) | (0.120) | (0.624) | (0.268) |
| Chronic diseases (ref: no) | 0.806^***^ | 1.011^*^ | 0.273 | 0.761^***^ | 1.084^**^ | 0.644^**^ |
|  | (0.259) | (0.564) | (0.197) | (0.118) | (0.540) | (0.266) |
| Self-rated health level=2 (ref: very healthy) | 0.307 | -0.390 | -0.917 | 0.332 | -0.832 | 0.349^**^ |
|  | (0.533) | (1.527) | (0.596) | (0.237) | (1.628) | (0.530) |
| Self-rated health level=3 (ref: very healthy) | 1.034^**^ | 0.619 | 0.616^***^ | 0.692^***^ | 1.058 | 1.153^**^ |
|  | (0.485) | (1.458) | (0.554) | (0.217) | (1.523) | (0.485) |
| Self-rated health level=4 (ref: very healthy) | 1.402^***^ | -0.855 | 0.191 | 0.973^***^ | -0.206 | 1.293^**^ |
|  | (0.518) | (1.496) | (0.560) | (0.233) | (1.537) | (0.521) |
| Self-rated health level=5 (ref: very healthy) | 3.498^***^ | 2.035 | 0.441^***^ | 1.486^***^ | 2.412 | 3.443^***^ |
|  | (0.523) | (1.454) | (0.126) | (0.236) | (1.500) | (0.530) |
| Type of medical insurance=1 (ref: none) | -0.691 | 2.841 | -0.808 | 0.522^**^ | 3.408^*^ | -0.701 |
|  | (0.549) | (1.934) | (0.705) | (0.248) | (1.936) | (0.554) |
| Type of medical insurance=2 (ref: none) | -1.554 | 1.927 | 0.085 | 0.467 | 0.642 | -1.126 |
|  | (1.074) | (3.221) | (1.162) | (0.493) | (3.188) | (1.100) |
| Type of medical insurance=3 (ref: none) | -2.391^**^ | -0.983 | 0.365 | 1.389^***^ | -1.700 | -2.202^*^ |
|  | (1.129) | (2.538) | (0.955) | (0.505) | (2.621) | (1.129) |
| Type of medical insurance=4 (ref: none) | -1.419 | -3.622 | -1.112 | 0.973 | -0.498 | -0.603 |
|  | (1.877) | (4.470) | (1.571) | (0.882) | (4.313) | (1.970) |
| Type of medical insurance=5 (ref: none) | -1.579 | 0.341 | -0.726 | 0.710 | -1.337 | -1.264 |
|  | (1.316) | (2.738) | (1.083) | 0.590 | (2.972) | (1.317) |
| Age | 0.352 | -0.260 | 0.138 | 0.141 | 0.079 | -0.234 |
|  | (0.260) | (0.609) | (0.216) | (0.120) | (0.592) | (0.267) |
| Gender (ref: female) | -1.075 | 3.947 | -1.553 | 4.951^**^ | 6.533 | -1.845 |
|  | (4.892) | (8.493) | (3.093) | (2.122) | (8.490) | (4.741) |
| Education level 2 (ref: Education level 1) | -1.446 | 1.253 | 0.996 | 0.397 | 1.680 | -2.014 |
|  | (1.341) | (2. 764) | (0.950) | (0.616) | (2.608) | (1.376) |
| Education level 3 (ref: Education level 1) | 2.572 | 2.363 | 0.016 | -0.012 | 1.343 | 2.322 |
|  | (3.117) | (3.011) | (1.235) | (1.353) | (2.605) | (3.021) |
| Ln (Per capita family income) | -0.385^***^ | -0.847^***^ | 0.031 | 0.165^***^ | -0.397 | -0.376^***^ |
|  | (0.130) | (0.270) | (0.095) | (0.060) | (0.260) | (0.133) |
| Constant | -0.596 | 30.419 | -6.295 | -9.903 | 9.353 | 36.163^**^ |
|  | (17.307) | (40.286) | (14.396) | (7.938) | (39.506) | (17.725) |
| Regional fixed effect | Yes | Yes | Yes | Yes | Yes | Yes |
| Time fixed effect | Yes | Yes | Yes | Yes | Yes | Yes |
| R-squared | 0.546 | 0.631 | 0.825 | 0.055 | 0.624 | 0.548 |

Note: Standard errors in parentheses, ^*^, ^**^, ^***^ represent the significance level of 10%, 5%, and 1%, respectively

**Supplementary Table 2** Robustness test (replace explanatory variables) for intergenerational support and medical expenditure on the depression of the rural elderly

| Variables | Model 1 | Model 2 | Model 3 | Model 4 |
| --- | --- | --- | --- | --- |
|  | Depression | Inpatient | Non-inpatient | Depression |
| The amount of economic support | -0.039 | -0.041 | 0.084^***^ | -0.061 |
|  | (0.048) | (0.040) | (0.022) | (0.118) |
| The frequency of care support | 0.022 | 0.064^**^ | 0.024 | 1.147^*^ |
|  | (0.038) | (0.030) | (0.017) | (0.088) |
| Emotional support | -0.871^***^ | 0.025 | 0.030 | -1.118^***^ |
|  | (0.174) | (0.149) | (0.120) | (0.439) |
| Ln (inpatient medical expenditure) |  |  |  | -0.200 |
|  |  |  |  | (0.285) |
| Ln (Non-inpatient medical expenditure) |  |  |  | 0.323^***^ |
|  |  |  |  | (0.106) |
| Number of children | -0.493^*^ | -0.369 | 0.036 | 0.337 |
|  | (0.267) | (0.228) | (0.120) | (0.653) |
| Chronic diseases (ref: no) | 0.791^***^ | 0.287 | 0.731^***^ | 1.063^*^ |
|  | (0.260) | (0.197) | (0.118) | (0.587) |
| Self-rated health level=2 (ref: very healthy) | 0.321 | -0.958 | 0.344 | -1.064 |
|  | (0.535) | (0.593) | (0.237) | (1.670) |
| Self-rated health level=3 (ref: very healthy) | 1.078^**^ | -0.578 | 0.675^***^ | 0.565 |
|  | (0.487) | (0.553) | (0.216) | (1.590) |
| Self-rated health level=4 (ref: very healthy) | 1.391^***^ | 0.294 | 0.977^***^ | -0.767 |
|  | (0.520) | (0.555) | (0.232) | (1.608) |
| Self-rated health level=5 (ref: very healthy) | 3.486^***^ | 0.454 | 1.483^***^ | 1.695 |
|  | (0.525) | (0.544) | (0.235) | (1.580) |
| Type of medical insurance=1 (ref: none) | -0.569 | -0.934 | 0.493^**^ | 3.208^*^ |
|  | (0.550) | (0.706) | (0.247) | (2.118) |
| Type of medical insurance=2 (ref: none) | -1.424 | 0.151 | 0.452 | 0.763 |
|  | (1.077) | (1.164) | (0.491) | (3.389) |
| Type of medical insurance=3 (ref: none) | -2.400^**^ | 0.333 | 1.329^***^ | -1.796 |
|  | (1.133) | (0.959) | (0.504) | (2.744) |
| Type of medical insurance=4 (ref: none) | -1.203 | -0.872 | 1.071 | 0.161 |
|  | (1.885) | (1.581) | (0.881) | (5.022) |
| Type of medical insurance=5 (ref: none) | -1.590 | -0.783 | 0.663 | -1.566 |
|  | (1.321) | (1.087) | 0.588 | (3.033) |
| Age | 0.350 | 0.166 | 0.126 | -0.209 |
|  | (0.261) | (0.217) | (0.119) | (0.603) |
| Gender (ref: female) | -0.492 | -0.523 | 5.026^**^ | 5.200 |
|  | (4.907) | (3.090) | (2.115) | (8.397) |
| Education level 2 (ref: Education level 1) | -1.557 | 0.697 | 0.469 | 0.931 |
|  | (1.345) | (0.944) | (0.615) | (2.886) |
| Education level 3 (ref: Education level 1) | 2.521 | 0.032 | 0.138 | 1.332 |
|  | (3.128) | (1.237) | (1.350) | (2.650) |
| Ln (Per capita family income) | -0.404^***^ | 0.019 | 0.156^***^ | -0.725 |
|  | (0.130) | (0.095) | (0.060) | (0.288) |
| Constant | -0.786 | -8.137 | -8.783 | 29.943 |
|  | (17.374) | (14.492) | (7.920) | (40.241) |
| Regional fixed effect | Yes | Yes | Yes | Yes |
| Time fixed effect | Yes | Yes | Yes | Yes |
| R-squared | 0.543 | 0.824 | 0.061 | 0.625 |

Note: Standard errors in parentheses, ^*^, ^**^, ^***^ represent the significance level of 10%, 5%, and 1%, respectively

**Supplementary Table 3** The number of the observations regarding the PSM test

| Treatment variables | Outcome variables | Treatment assignment | Number of observations (N) | | | |
| --- | --- | --- | --- | --- | --- | --- |
|  |  |  | Off support | | On support | Total |
| Economic support | Depression | Untreated | 7 | | 598 | 1077 |
|  |  | Treated | 8 | | 464 |  |
| Care support |  | Untreated | 13 | | 841 | 1053 |
|  |  | Treated | 2 | | 197 |  |
| Emotional support |  | Untreated | 0 | | 61 | 3948 |
|  |  | Treated | 114 | | 3773 |  |
| Non-inpatient medical expenditure |  | Untreated | 6 | | 910 | 4497 |
|  |  | Treated | 41 | | 3540 |  |
| Economic support | Inpatient medical expenditure | Untreated | 7 | 682 | | 1239 |
|  |  | Treated | 6 | 544 | |  |
| Care support |  | Untreated | 13 | 957 | | 1212 |
|  |  | Treated | 2 | 240 | |  |
| Economic support | Non-inpatient medical expenditure | Untreated | 10 | 595 | | 1077 |
|  |  | Treated | 5 | 467 | |  |
| Care support |  | Untreated | 18 | 836 | | 1052 |
|  |  | Treated | 0 | 199 | |  |
